# Dihydropyridines Allosterically Modulate Hsp90 Providing a Novel Mechanism for Heat Shock Protein Co-induction and Neuroprotection

**DOI:** 10.3389/fmolb.2018.00051

**Published:** 2018-06-07

**Authors:** Mark S. Roe, Ben Wahab, Zsolt Török, Ibolya Horváth, László Vigh, Chrisostomos Prodromou

**Affiliations:** ^1^Genome Damage and Stability Centre, University of Sussex, Brighton, United Kingdom; ^2^Sussex Drug Discovery Centre, University of Sussex, Brighton, United Kingdom; ^3^Institute of Biochemistry, Biological Research Centre, Hungarian Academy of Sciences (HAS), Szeged, Hungary

**Keywords:** Alzheimer's disease, dementia, neuroprotection, heat shock proteins, dihydropyridine compounds, heat shock response, Hsp90

## Abstract

Chaperones play a pivotal role in protein homeostasis, but with age their ability to clear aggregated and damaged protein from cells declines. Tau pathology is a driver of a variety of neurodegenerative disease and in Alzheimer's disease (AD) it appears to be precipitated by the formation of amyloid-β (Aβ) aggregates. Aβ-peptide appears to trigger Tau hyperphosphorylation, formation of neurofibrillary tangles and neurotoxicity. Recently, dihydropyridine derivatives were shown to upregulate the heat shock response (HSR) and provide a neuroprotective effect in an APPxPS1 AD mouse model. The HSR response was only seen in diseased cells and consequently these compounds were defined as co-inducers since they upregulate chaperones and co-chaperones only when a pathological state is present. We show for compounds tested herein, that they target predominantly the C-terminal domain of Hsp90, but show some requirement for its middle-domain, and that binding stimulates the chaperones ATPase activity. We identify the site for LA1011 binding and confirm its identification by mutagenesis. We conclude, that binding compromises Hsp90's ability to chaperone, by modulating its ATPase activity, which consequently induces the HSR in diseased cells. Collectively, this represents the mechanism by which the normalization of neurofibrillary tangles, preservation of neurons, reduced tau pathology, reduced amyloid plaque, and increased dendritic spine density in the APPxPS1 Alzheimer's mouse model is initiated. Such dihydropyridine derivatives therefore represent potential pharmaceutical candidates for the therapy of neurodegenerative disease, such as AD.

## Introduction

The chaperone network helps maintain protein homeostasis (proteostasis) by aiding protein folding, by clearing aggregated and damaged protein from cells and by maintaining proteins in an active state (Hartl, 1996; Kim et al., 2013; Penke et al., 2018). However, with age there is a decline in the capacity of cells to maintain proteostasis and the prevalence of neurodegenerative diseases such as Alzheimer's- (AD) and Parkinson's-disease increases (Valastyan and Lindquist, 2014; Toth et al., 2015; Hartl, 2017). Heat shock proteins have been shown to decrease age-related proteotoxicity, while simultaneously increasing the life span in organisms such as *Caenorhabditis elegans* (Murshid et al., 2013).

The current model of AD suggests that the overexpression of mutant forms of β-amyloid precursor protein (APP) lead to amyloid-β (Aβ) plaque and neurofibrillary tangle formation by Tau (Choi et al., 2015). It is thought that Aβ peptide triggers the hyperphosphorylation of Tau, a Hsp90 dependent process (Jinwal et al., 2011), that subsequently leads to neurofibrillary tangles and neurotoxicity (Hardy and Selkoe, 2002; Selkoe, 2002; Oddo et al., 2003; Tanzi and Bertram, 2005; Annaert and De Strooper, 2010; Karran et al., 2011; Benilova et al., 2012). Maintenance of healthy homeostasis of proteins by modulation of protein processing and folding systems by chaperone induction represents a prime focus for drug discovery with AD and neurodegenerative diseases as key therapeutic targets (Hamos et al., 1991; Alavez et al., 2011). The accumulation and aggregation of Tau is actually facilitated by Hsp90, thus increasing its toxicity (Blair et al., 2013). Hence, there has been a drive to develop Hsp90 inhibitors that promote the degradation of Tau (Dickey et al., 2007; Luo et al., 2007; Blair et al., 2014). Small molecules that interfere with the normal function of Hsp90 are known to induce the heat shock response (HSR) and increase the degradation of Hsp90 dependent client proteins such as Tau by a Hsp90-CHIP E3 ubiquitin ligase dependent pathway (Dickey et al., 2007).

The mechanism of neuroprotective action of dihydropyridine (DHP) derivatives remains elusive (Kasza et al., 2016), but it was recently reported that they could activate the HSR (Kasza et al., 2016). The HSR response was only seen in diseased cells and consequently these compounds were defined as co-inducers since they upregulate chaperones and co-chaperones only in the context of a pathological state (Kasza et al., 2016). Noteworthy, is that Hsp27 and Hsp70 levels are lower in the brain of AD patients and consequently the induction of heat shock proteins represents a potential strategy for the treatment of neurodegenerative disorders (Klettner, 2004). The induction of heat shock proteins, such as Hsp27 and Hsp72, that promote cell survival by preventing protein aggregation and promoting protein refolding and by eliciting protein degradation of aggregated proteins (Ehrnsperger et al., 1997), might represent a novel tool in the treatment of neurodegenerative disease (Söti and Csermely, 2002; Franklin et al., 2005; Morimoto, 2008; Manaenko et al., 2010; Tóth et al., 2013; Bobkova et al., 2014; Eroglu et al., 2014; Kalmar et al., 2014; Wang et al., 2014). This is all the timelier since the announcement of a variety of clinical trial failures including those for idalopirdine and intepirdine, selective 5-hydroxytryptamine-6 receptor antagonists, and solanezumab, an Aβ antibody (Atri et al., 2018; Honig et al., 2018; Simpson, 2018).

The inhibition of Hsp90 is best characterized by binding of small molecules to its N-terminal ATP binding domain, and include geldanamycin, radicicol, and synthetic molecules such as AUY922 (Roe et al., 1999; Brough et al., 2008). Less well characterized are the C-terminal Hsp90 binding compounds, including Novobiocin, Coumermycin and KU-32 (Marcu et al., 2000; Donnelly and Blagg, 2008; Kusuma et al., 2012), and recently some compounds have emerged as activators of the ATPase activity of Hsp90. Such compounds include rhamnoside that are thought to modulate the conformation of Hsp90, lowering transition states that favor N-terminal dimerization and therefore ATPase activity (Sattin et al., 2015). Rhamnoside, and its derivatives (Sattin et al., 2015), were selected initially based on the stereoelectronic properties displayed by an allosteric site in the C-terminal domain of Hsp90, which itself was identified using molecular dynamic simulations (Vettoretti et al., 2016). Rhamnoside derivatives, which have a benzofuran scaffold at their core, were later shown to accelerate the Hsp90 cycle and to stimulate the ATPase activity up to six-fold and to be cytotoxic against a variety of cancer cell lines (Sattin et al., 2015). Unlike N-terminal inhibitors, the benzofuran derivatives did not induce the HSR, but reduced the levels of Hsp90 dependent client proteins such as ErbB2, Akt, Cdk4, and Survivin (Sattin et al., 2015). In contrast to activators, a set of structurally-symmetrical chemical derivatives based on NSC145366, which contains a bisphenol A core, were shown to be allosteric inhibitors of the ATPase activity of Hsp90 (Goode et al., 2017). These derivatives compromised the chaperoning function of Hsp90, by inhibiting its ability to prevent the aggregation of citrate synthase and the refolding of luciferase. NSC145366 was particularly active in promoting androgen receptor and BRAC1 client protein degradation in the LNCaP androgen sensitive prostate adenocarcinoma cancer cell line. This inhibitor did not induce the classical HSR seen with N-terminal inhibitors. To date, and to the best of our knowledge, no experimentally determined structure of Hsp90 in complex with a C-terminal binding compound has been reported.

Here we show that DHP derivatives bind to the C-terminal- and middle-domain of Hsp90 and activate its ATPase activity, which in turn compromises the chaperone activity of Hsp90. Through docking studies and mutagenesis, we identify the C-terminal binding site of DHP derivatives. The molecular basis for the mechanism by which these DHP derivatives have a neuroprotective effect in the APPxPS1 Alzheimer's mouse model is proposed.

## Materials and methods

### Protein purification and synthesis of dihydropyridine derivatives

pRSETA was used to express yeast full–length Hsp90, human full-length Hsp90α, Hsp90β and a variety of yeast fragments: [Hsp90^1−220^ (N-terminal domain), Hsp90^1−560^ (N and middle-domain), Hsp90^273−709^ (middle and C-terminal domain), Hsp90^546−709^ (C-terminal domain), monomeric yeast Hsp90 (Hsp90^F675L, I679M^), and human Hsp90^9−236^ (N-terminal domain)]. Mutants of Hsp90 were generated by Genscript (860 Centennial Ave., Piscataway, NJ 08854, USA). Constructs were purified by Talon affinity, gel-filtration with an appropriate molecular mass exclusion, and ion-exchange chromatography as previously described (Panaretou et al., 1998). The synthesis of DHP derivatives was previously described (Kasza et al., 2016).

### Isothermal titration calorimetry

Heat of interaction was measured on an ITC200 microcalorimeter (Microcal) under the same buffer conditions (20 mM Tris, pH 7.5, containing 1 mM EDTA, and 5 mM NaCl) at 30°C. Either 1.9 or 3.8 μL aliquots of the LA1011, LA1014, or LA1026 [provided by Gedeon Richter Ltd. (www.richter.hu)] at 1 mM were injected into 60 μM of Hsp90 protein or a variety of constructs, including mutants of Hsp90 and complexes of Hsp90 with 5 mM ADP or AMPNP, or Hsp90 complexed with 5 mM AMPPNP and 600 μM Sba1. 6 mM MgCl_2_ was included in the buffer in experiments containing nucleotide. Additionally, 3.8 μL aliquots of 90 μM Sti1 were injected into 6 μM Hsp90 containing 1 mM LA1011. 1 mM LA1011 was also injected into 60 μM human Hsp72. The concentration of components used in ITC experiments were based on previous experiments showing how Hsp90 interacts with nucleotide, Sba1 and Sti1 (Siligardi et al., 2004; Prodromou et al., 2009; Vaughan et al., 2009). Heats of dilution were determined by diluting injectant at 30°C into buffer. Data were fitted using a non-linear least squares curve-fitting algorithm (Microcal Origin) with three floating variables: stoichiometry, binding constant (*K*_B_ = 1/*K*_D_) and change of enthalpy of interaction (Δ*H*°).

### ATPase assays and gel-filtration

ATPase assays using 2 μM Hsp90 were conducted as previously described (Prodromou et al., 1999) and displayed a turnover of 1.2 per min. Geldanamycin was prepared at 20 mM in DMSO, whereas 50 mM LA1011, LA1014, and LA1026 were soluble in 20 mM Tris pH7.5, 1 mM EDTA, and 5 mM NaCl. Estimation of the relative molecular mass of wild type Hsp90 and the monomeric yeast Hsp90^F675L, I679M^ was conducted on a superpose 6 10/300 GL gel-filtration column equilibrated in 20 mM Tris pH 7.5, 1 mM EDTA, 150 mM NaCl, and 1 mM DDT.

### Citrate synthase aggregation assay

150 nM citrate synthase (2 mg/mL), was incubated at 45°C for 60 min in 1 mL of 90 mM HEPES buffer containing 20 mM KOH, 50 mM KCI, 10 mM (NH_4_)_2_SO_4_, 2 mM potassium acetate, pH 7.8, and Hsp90 at 0, 120, 240, 480, 600, or 1000 nM. Experiments using 0.1 or 0.2 mM LA1011 were conducted with 600 nM Hsp90. The change in optical density was monitored at 320 nm.

### Refolding of denatured luciferase

QuantiLum Luciferase (Promega E170A) was diluted to 100 ug/mL in 25 mM Tricine pH 7.8, containing 10 mM MgCl_2_, 1 mM DTT, 0.1 mM EDTA, 10% glycerol, and 100 mg/mL BSA (sigma 05470) and denatured at 44°C for 30 min. 0.5 μL of denatured luciferase was then added to 20 μL of rabbit reticulocyte lysate (Promega, L415A) containing either buffer or LA1011 at 1 or 5 mM or geldanamycin at 2.5 or 5 μM and incubated at 30°C. Geldanamycin was prepared in DMSO at 0.5 and 2 mM and geldanamycin experiments contained a final DMSO concentration of 1%. Refolding of luciferase was determined by removing 2 μL aliquots of the rabbit reticulocyte mix at 0, 15, 30 45, 60, 90, and 120 min and adding this to 48 μL of Briglt-Glo^TM^ reconstituted reagent (Promega, E2610), which contains buffer, substrate and ATP required for luciferase activity, as instructed by the manufacturer. Luminescence was measured in a CLARIOstar^R^ microplate reader using half area 96 well flat bottom plates.

### Structural modeling and biomolecular simulations

HSP90 crystal structures were analyzed and one, an Hsp90α structure, was selected for docking, based on the regions required for HSP90 homodimerization (PDB code 3Q6N, 3.05A) (Lee et al., 2011). The structure was prepared in Schrodinger Suite 2017-3 (Schrodinger LLC, Cambridge, MA 02142, USA) at pH 7.4 before running a site finding scan. Structure-Activity Relationship (SAR) and Site Directed Mutagenesis (SDM) data were used to narrow down the binding pocket, which was then used as a starting locus for Induced Fit Docking (Glide + Prime IFD, OPLS 3.0, XP). The Ligand (LA1011) was prepared with LigPrep at pH 7.4, and a single low energy conformer was presented for docking. The docking results were compared to experimental SAR and SDM data and the most reasonable pose was checked for stability in Molecular Dynamics (Desmond, 100 ns NPT, SPC, 300 K). Binding mode analyses were conducted on the outcomes of the molecular dynamics simulations. PyMol was used to create cartoon images of the structures^1^.

## Results

### Hsp90 is the molecular target of dihydropyridine derivatives

**Figure 1:**
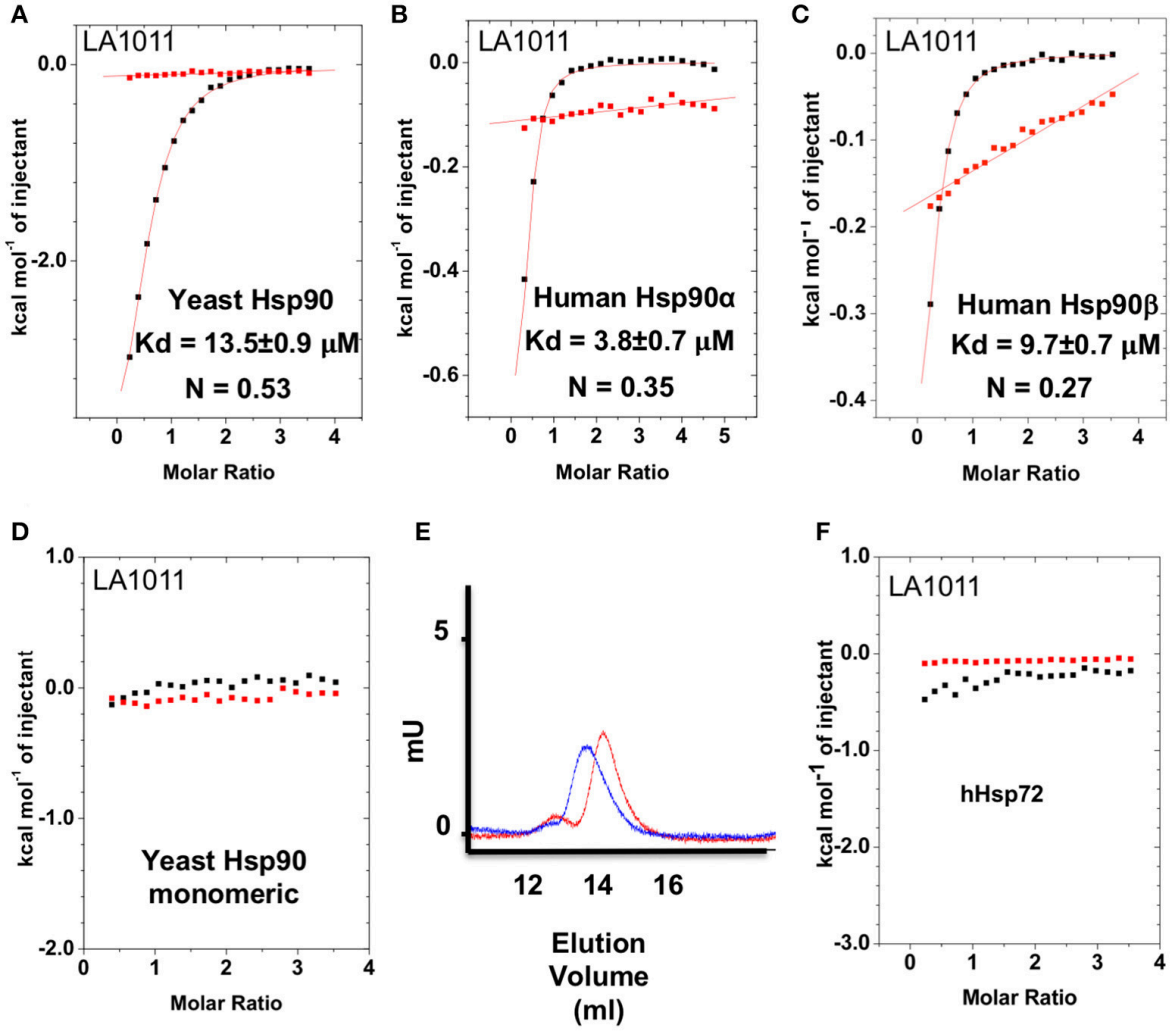
Isothermal titration curves for the binding of LA1011 to a variety of intact chaperone constructs and gel-filtration analysis of Hsp90 monomeric mutant. **(A)** yeast full-length; **(B)** human Hsp90α full-length; **(C)** human Hsp90β full-length; and **(D)** yeast monomeric Hsp90^F675L, I679M^. **(E)** Gel-filtration profile of wild type dimeric yeast Hsp90 (blue trace, peak elution volume 13.8 ml) and the monomeric mutant Hsp90^F675L, I679M^ (red trace, peak elution volume 14.4 ml), showing a decrease in relative molecular mass of the monomeric mutant Hsp90^F675L, I679M^ mutant. **(F)** LA1011 binding to human Hsp72. Red lines in ITC figures represent heat of dilution, while the black fittings represent the corrected heat of interaction for the experiment.

**Figure 2:**
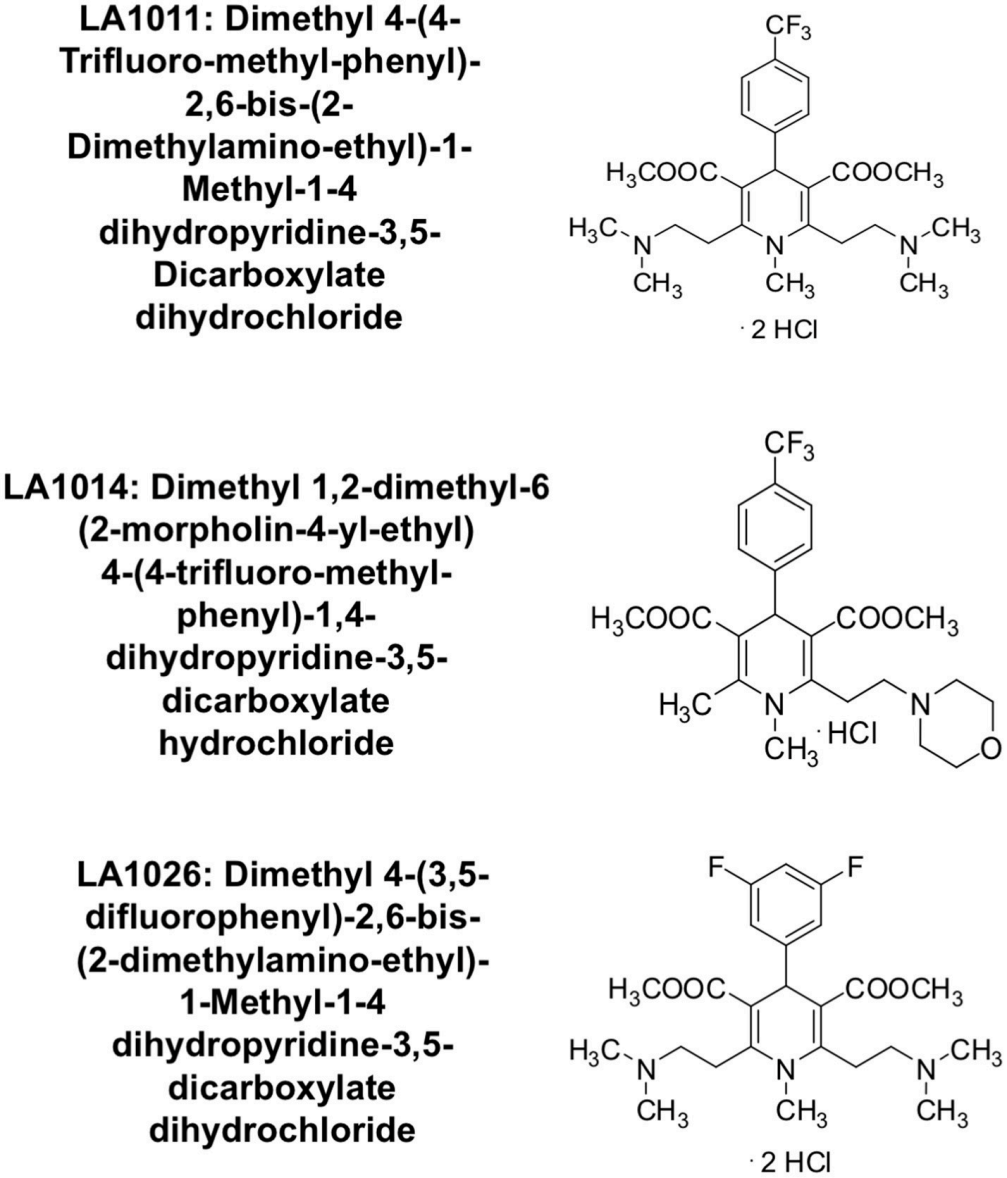
Chemical structure of the synthesized 1,4-dihydropyridine derivatives.

The upregulation of Hsp27, Hsp40 and Hsp70 is a well-documented response to the perturbation of Hsp90 activity, resulting from the activation of HSF1 (Heimberger et al., 2013). We therefore used isothermal titration calorimetry (ITC) to investigate whether the water-soluble LA1011 drug candidate would bind Hsp90, thus providing a route by which such compounds are able to co-induce the HSR. We found that LA1011 bound to yeast Hsp90 (*K*d = 13.5 ± 0.9 μM), human Hsp90α (*K*d = 3.8 ± 0.7 μM), and human Hsp90β (*K*d = 9.7 ± 0.7 μM) (Figures 1A–C). LA1011 bound Hsp90 with a small favorable enthalpic and entropic contribution in all cases (Table 1). The stoichiometry of binding appeared to be one molecule of LA1011 bound too one Hsp90 dimer, indicating that binding must occur at a symmetrically located position along the central long-axis of Hsp90, and it is noteworthy that LA1011 possesses a plane of symmetry (Figure 2). The degree of symmetrical interaction with each Hsp90 protomer is, however, not absolute since LA1014 lacks the symmetry of LA1011, but non-the-less still bound Hsp90.

**Table 1:** Thermodynamic terms for the binding of dihydropyridine derivatives to Hsp90 constructs.

**Protein**	**Compound**	**ΔH (cal/mol)**	**ΔS (cal/mol/Deg)**
Yeast Hsp90	LA1011	−4863 ± 194	6.24
Hsp90α	LA1011	−792 ± 80.7	22.2
Hsp90β	LA1011	−654.9 ± 43.5	20.8
Yeast 273–709	LA1011	−5110 ± 187	6.07
Yeast 546–709	LA1011	−2603 ± 706	10.7
Yeast 273–709	LA1014	−871.4 ± 145	18.6
Yeast 273–709	LA1026	−1233 ± 210	15.7

*Yeast 273–709 represents the middle and C-terminal domains of yeast Hsp90, while Yeast 546–709 represents the yeast C-terminal domain alone*.

To ascertain the stoichiometry of the reaction we investigated the binding of LA1011 to a monomeric mutant of yeast Hsp90, that contains two mutations, F675L and I679M that disrupt dimerization of the C-terminal domains, and no binding was detected (Figure 1D). We confirmed that this mutant was indeed monomeric by gel-filtration chromatography (Figure 1E). This confirmed that LA1011 only binds dimeric Hsp90 and that one molecule of LA1011 must simultaneously bind to both halves of the Hsp90 molecule, although the degree of interaction with each protomer remains unknown. Finally, we tested the ability of LA1011 to bind human Hsp72 and found that it did not interact with this chaperone, suggesting that DHP compounds specifically target the Hsp90 chaperone system (Figure 1F).

### LA1011 binds the C-terminal domain of Hsp90

**Figure 3:**
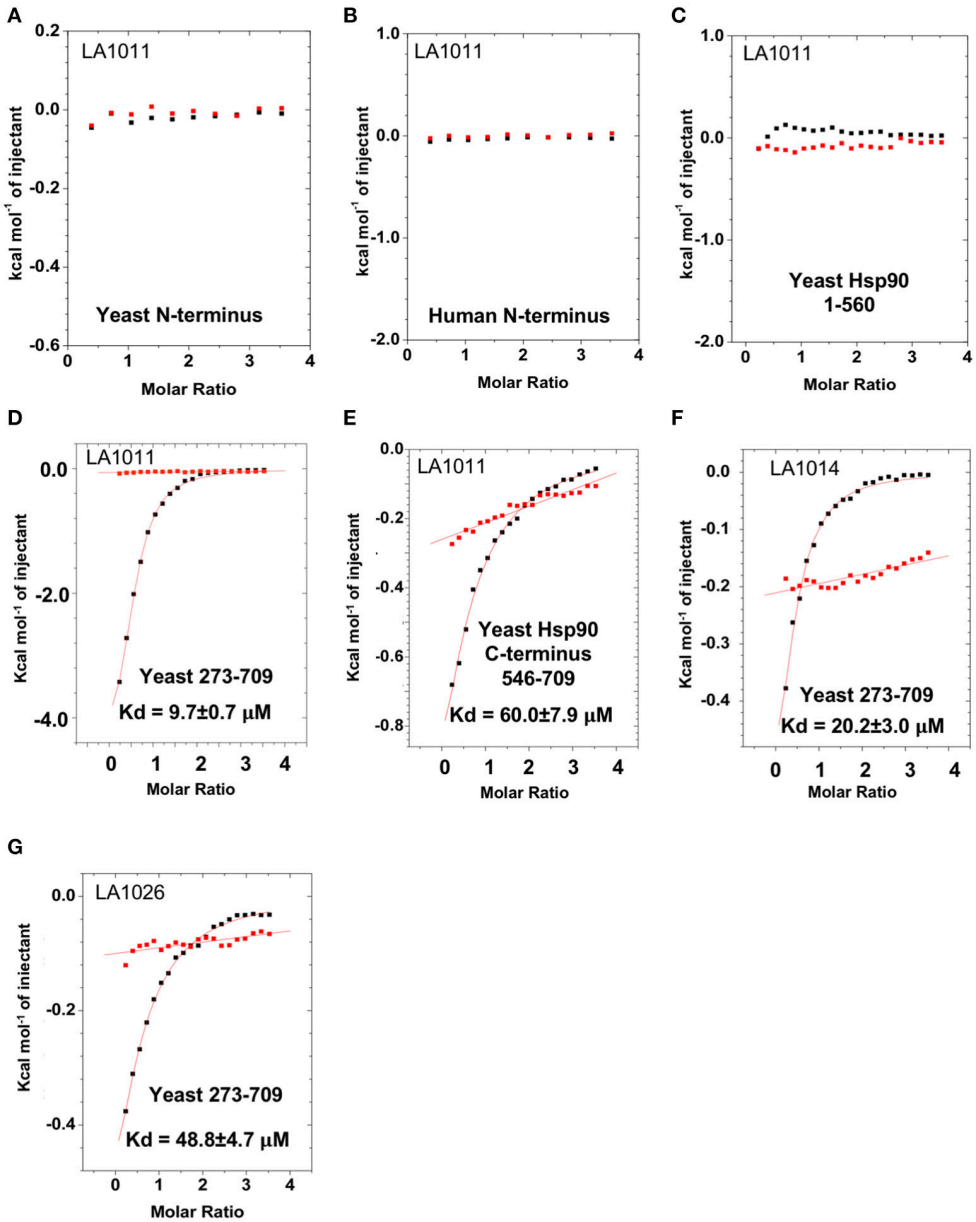
Isothermal titration curves for the binding of DHPs to a variety of truncated Hsp90 constructs. Binding of LA1011 to **(A)** yeast Hsp90 N-terminal domain; **(B)** Hsp90α N-terminal domain; **(C)** Hsp90^1−560^ (N and middle domain); **(D)** Hsp90^273−709^ (middle and C-terminal domain); and **(E)** Hsp90^546−709^ (C-terminal domain). **(F)** The binding of LA1014 and **(G)** LA1026 to the Hsp90^273−709^ (middle and C-terminal domain). The C-terminal domain appears to be the main site of interaction for all DHP compounds tested. Red lines in ITC figures represent heat of dilution, while the black fittings represent the corrected heat of interaction for the experiment.

To determine the exact Hsp90 domain responsible for the binding of LA1011 we investigated binding to a variety of Hsp90 constructs, including the N-terminal domain of yeast Hsp90 and Hsp90α, Hsp90^1−560^ (representing the N- and middle-domain of yeast Hsp90), Hsp90^273−709^ (representing the middle- and C-domain of yeast Hsp90), and finally a yeast C-terminal construct, Hsp90^546−709^. LA1011 failed to bind the isolated N-terminal domains of yeast and human Hsp90α and a construct (Hsp90^1−560^) representing the N- and middle-domain of yeast Hsp90 (Figures 3A–C). In contrast, LA1011 bound with the same stoichiometry and with a similar affinity to Hsp90^273−709^, representing the middle and C-terminal domain construct of yeast Hsp90 (*K*d = 9.7 ± 0.7 μM; Figure 3D), as with that seen for the intact yeast Hsp90 (*K*d = 13.5 ± 0.9 μM). The binding affinity was somewhat lower toward the yeast C-terminal domain alone (Hsp90^546−709^; *K*d = 60.6 ± 7.9 μM) (Figure 3E). The results suggest a single binding site located predominantly in the C-terminus of Hsp90, which is sufficient for the binding of LA1011, but that binding is favored in the presence of the middle domain. Similarly, LA1014 and LA1026 (Figures 3F,G) bound yeast Hsp90^273−709^, but with slightly lower affinity (*K*d = 20.2 ± 3.0 and 48.8 ± 4.7 μM, respectively). LA1014 and LA1026 also bound Hsp90^273−709^ displaying a small favorable enthalpy and entropy (Table 1).

### Nucleotide effects and co-chaperone binding

**Figure 4:**
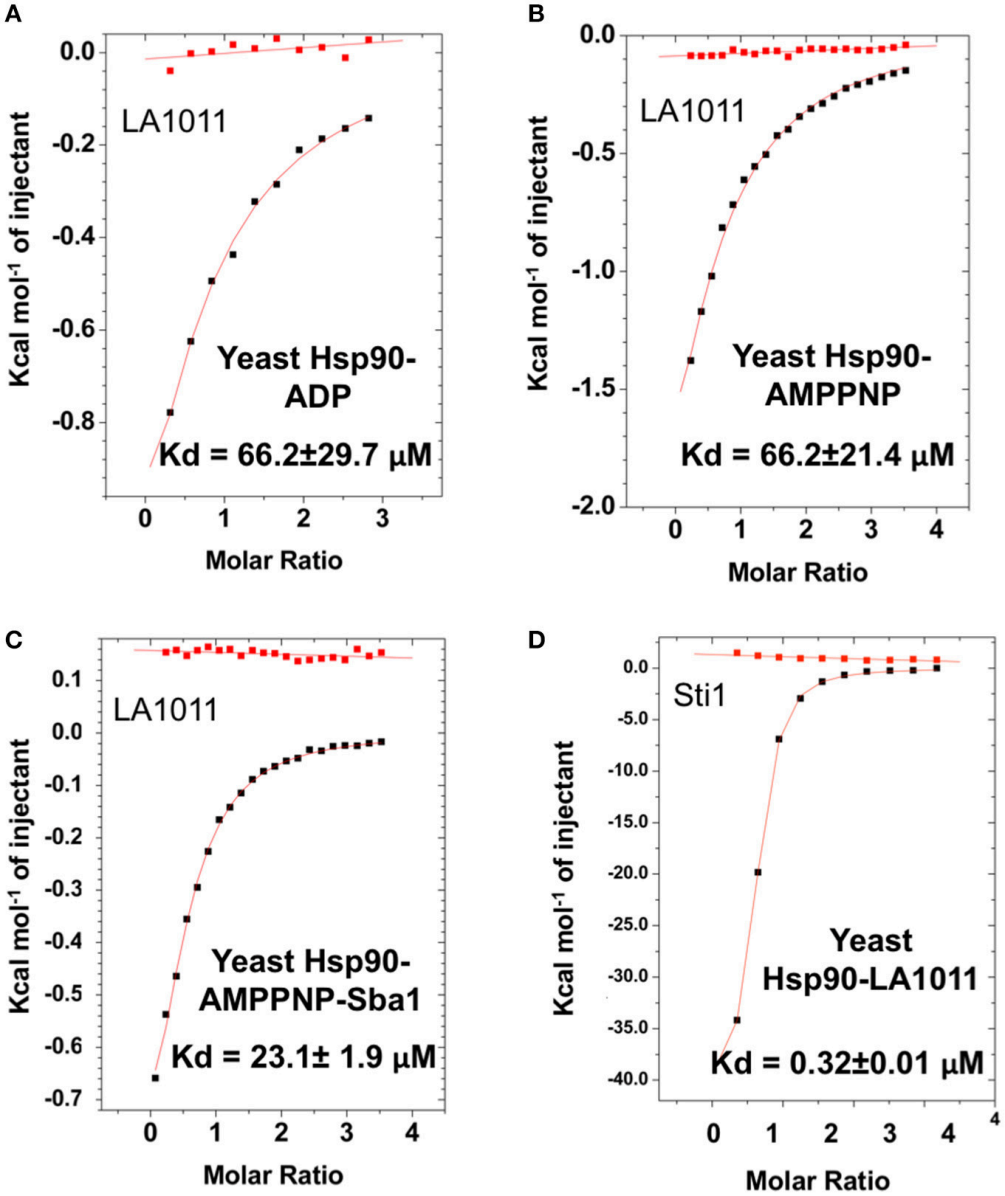
Effect of nucleotide on LA1011 binding and effects on or with co-chaperone interaction. Binding of LA1011 to **(A)** yeast Hsp90-ADP complex; **(B)** yeast Hsp90–AMPPNP complex, and **(C)** Hsp90-AMPPNP-Sba1 complex. **(D)** Binding of Sti1 to Hsp90-LA1011 complex. Nucleotide appears to decrease the affinity for LA1011, while LA1011 has little effect on Sti1 binding itself, based on previous studies (Prodromou et al., 1999). Sba1 weakens LA1011 binding slightly. Red lines in ITC figures represent heat of dilution, while the black fittings represent the corrected heat of interaction for the experiment.

We next tested the ability of LA1011 to bind intact yeast Hsp90 in the presence of ADP or AMPPNP, a non-hydrolysable analog of ATP. We found that both nucleotides compromised LA1011 binding, reducing the *K*d to approximately 66.2 μM (Figures 4A,B). Interestingly, using a Hsp90-AMPNP-Sba1 complex slightly weakened the binding of LA1011 (*K*d = 23.1 ± 1.9 μM; Figure 4C), relative to wild type yeast Hsp90. Finally, binding of the yeast co-chaperone Sti1 to Hsp90 was not compromised by LA1011 (*K*d = 0.32 ± 0.01 μM) (Figure 4D), when compared to previous studies showing that Sti1 binds yeast Hsp90 with a similar affinity (*K*d = 0.33 ± 0.03 μM) (Prodromou et al., 1999). These results indicate that both the closed (AMPPNP bound) and open (Sti1 bound) conformation of Hsp90 interacts with LA1011.

### LA1011 is an activator of the ATPase activity of Hsp90

**Figure 5:**
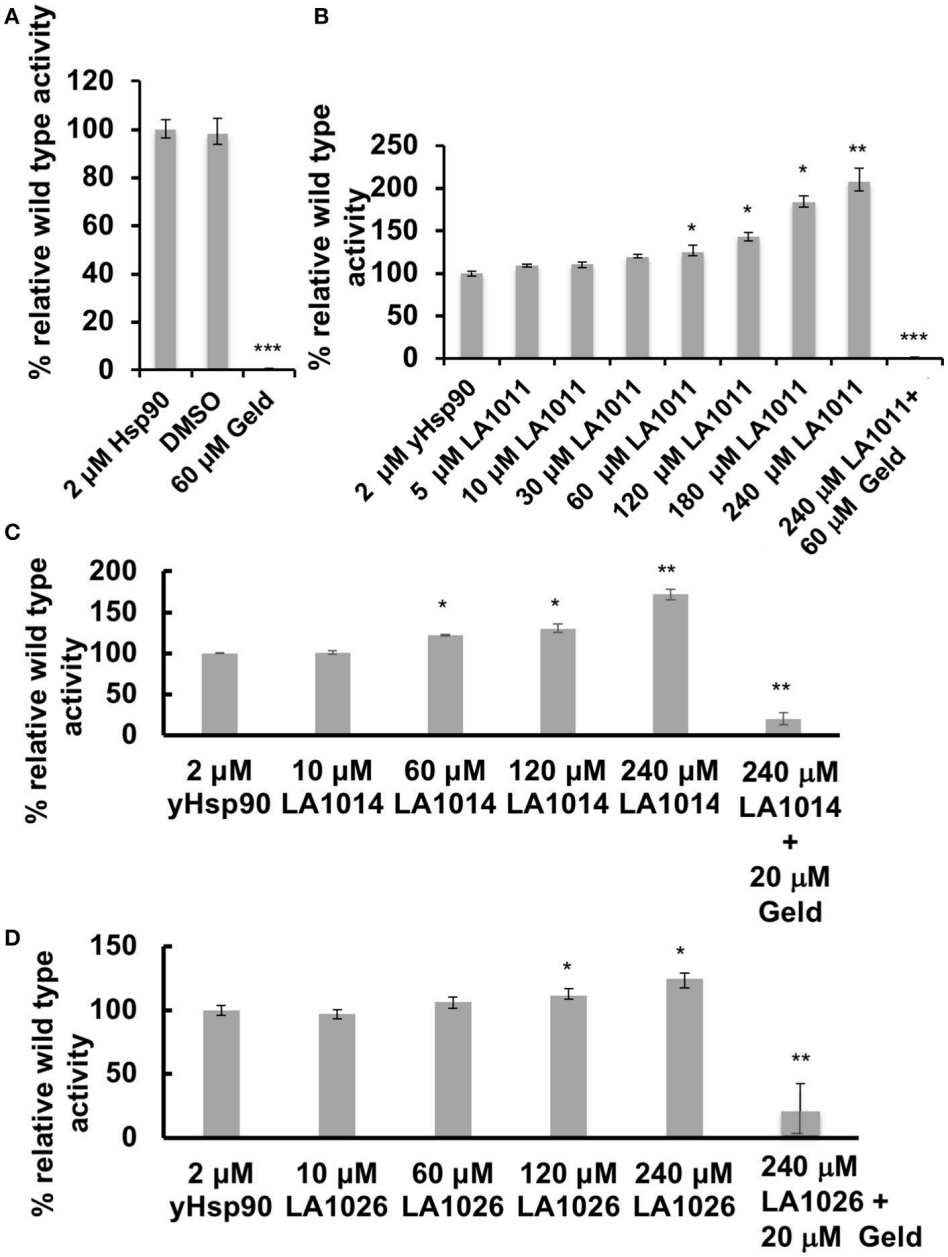
ATPase assays showing the activation of Hsp90 by DHP derivatives. The turnover for Hsp90 was 1.2 per min. **(A)** control assay showing the inhibition of yeast Hsp90 by geldanamycin (Geld). Activation of Hsp90 ATPase activity by **(B)** LA1011; **(C)** LA1014, and **(D)** LA1026. The activated activity can be inhibited by geldanamycin showing that activation of Hsp90 is specific. Error bars represent standard deviation and assays were carried out in triplicate. Paired t-test was used for statistical difference. *P ≤ 0.05, **P ≤ 0.01, and ***P ≤ 0.001.

The inhibition of the ATPase activity of Hsp90 by naturally occurring antibiotics and synthetic molecules is well documented (Roe et al., 1999; Zhao et al., 2012) and is a known mechanism for the upregulation of the HSR. Previous attempts to look at the effects of DHP derivatives on the ATPase activity of Hsp90 suggested that such derivatives did not target Hsp90 (Kasza et al., 2016). However, the levels of DHP derivatives previously used were below the *K*d values determined in this study. With this in mind we tested the effect of these compounds on their ability to modulate the ATPase activity of Hsp90. Control reactions with geldanamycin showed inhibition of Hsp90 ATPase activity (non-inhibited turnover 1.2 min), as expected (Figure 5A). In contrast, LA1011, LA1014, and LA1026 activated the ATPase activity of yeast Hsp90 (Figures 5B–D). These results suggest that the ATPase activity of Hsp90 can be allosterically modulated by the binding of a small molecule to the C-terminal domains of the chaperone.

### LA1011 compromises chaperoning by Hsp90

**Figure 6:**
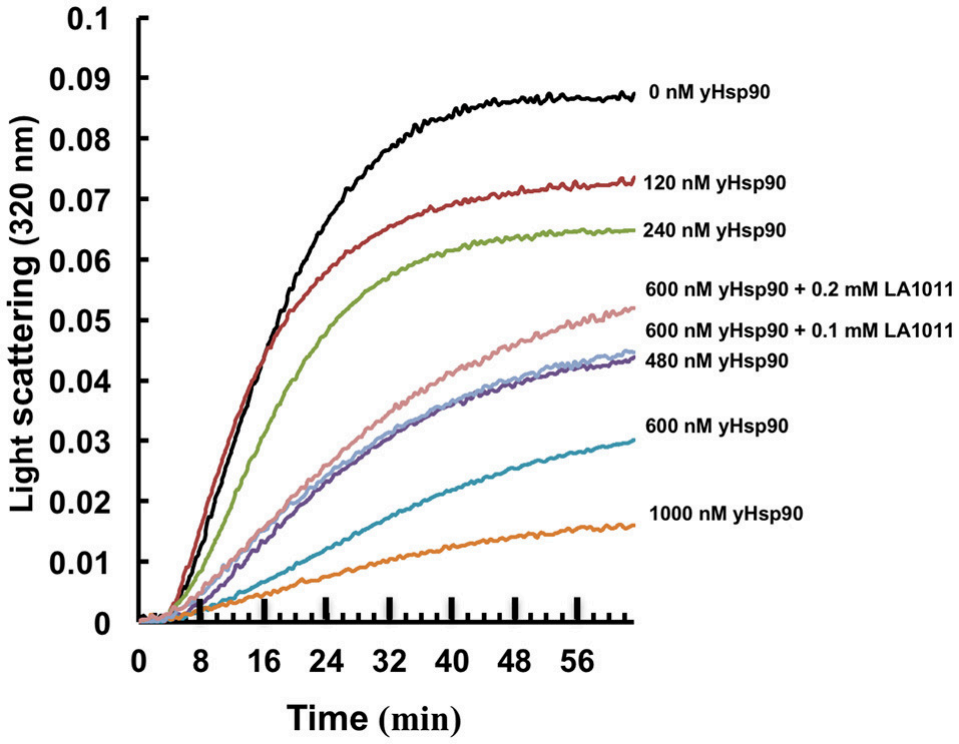
Citrate synthase aggregation assays. Increasing concentrations of yeast Hsp90 prevent citrate synthase aggregation at 45°C and this activity is compromised in the presence of LA1011.

It has been previously proposed that the C-terminal domain of Hsp90 is a potential interaction site for client proteins (Harris et al., 2004). Consequently, DHP derivatives have the potential to affect the ability of Hsp90 to chaperone such clients. We therefore investigated whether LA1011 would compromise Hsp90 in preventing the aggregation of citrate synthase (Figure 6). Based on the *K*d of 13 μM for the binding of LA1011 to the yeast protein the fractional occupancy for the binding of LA1011 would be 88–93%, calculated as [ligand]/[ligand]+*K*d, at 0.1–0.2 mM LA1011, respectively. At these concentrations, LA1011 disrupted yeast Hsp90's ability to protect citrate synthase from aggregation by around 50% at 45°C. This suggests that LA1011 might prevent access of client protein to Hsp90 or influence its ability to protect unfolding protein and so interferes with its chaperone activity.

### LA1011 inhibits the reactivation of denatured luciferase

**Figure 7:**
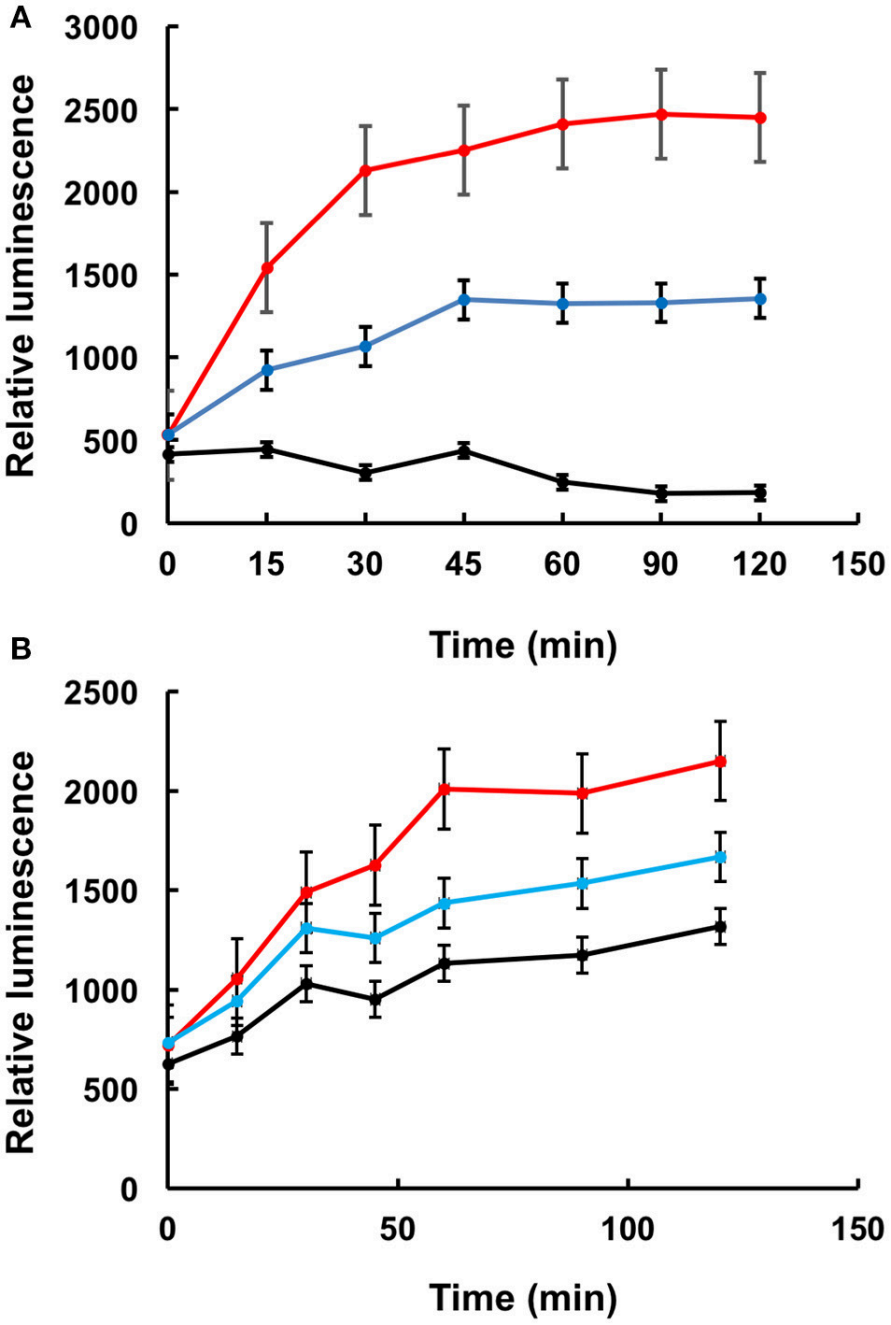
Refolding of luciferase. Refolding of luciferase in rabbit reticulocyte lysate is compromised in the presence of **(A)** LA1011 and **(B)** geldanamycin. Red, control without LA1011, or geldanamycin, blue, 1 mM LA1011, or 2 μM geldanamycin and black 5 mM LA1011 or 5 μM geldanamycin. Errors bars represent standard error and the experiment was carried out in triplicate.

To further confirm that LA1011 compromises Hsp90's ability to chaperone client protein we used luciferase refolding in rabbit reticulocyte lysates to investigate its ability to refold client protein. Based on the *K*d of 66 μM for the binding of LA1011 to Hsp90 in complex with AMPPNP, the fractional occupancy for LA1011 would be 93%, calculated as [ligand]/[ligand] + *K*d, at 1 mM LA1011. At this concentration, LA1011 inhibited Hsp90 by around 50% and 5 mM completely prevented refolding of luciferase confirming that LA1011 compromised the chaperoning function of Hsp90 (Figure 7A). In control assays using geldanamycin we found that 5 μM inhibited luciferase refolding by approximately 50% (Figure 7B). These results are consistent with LA1011 and geldanamycin disrupting the refolding of luciferase by either directly competing with luciferase binding to Hsp90 or through perturbation of the Hsp90 chaperone cycle.

### Structural modeling by docking and molecular dynamics reveals the C-terminal binding site

**Figure 8:**
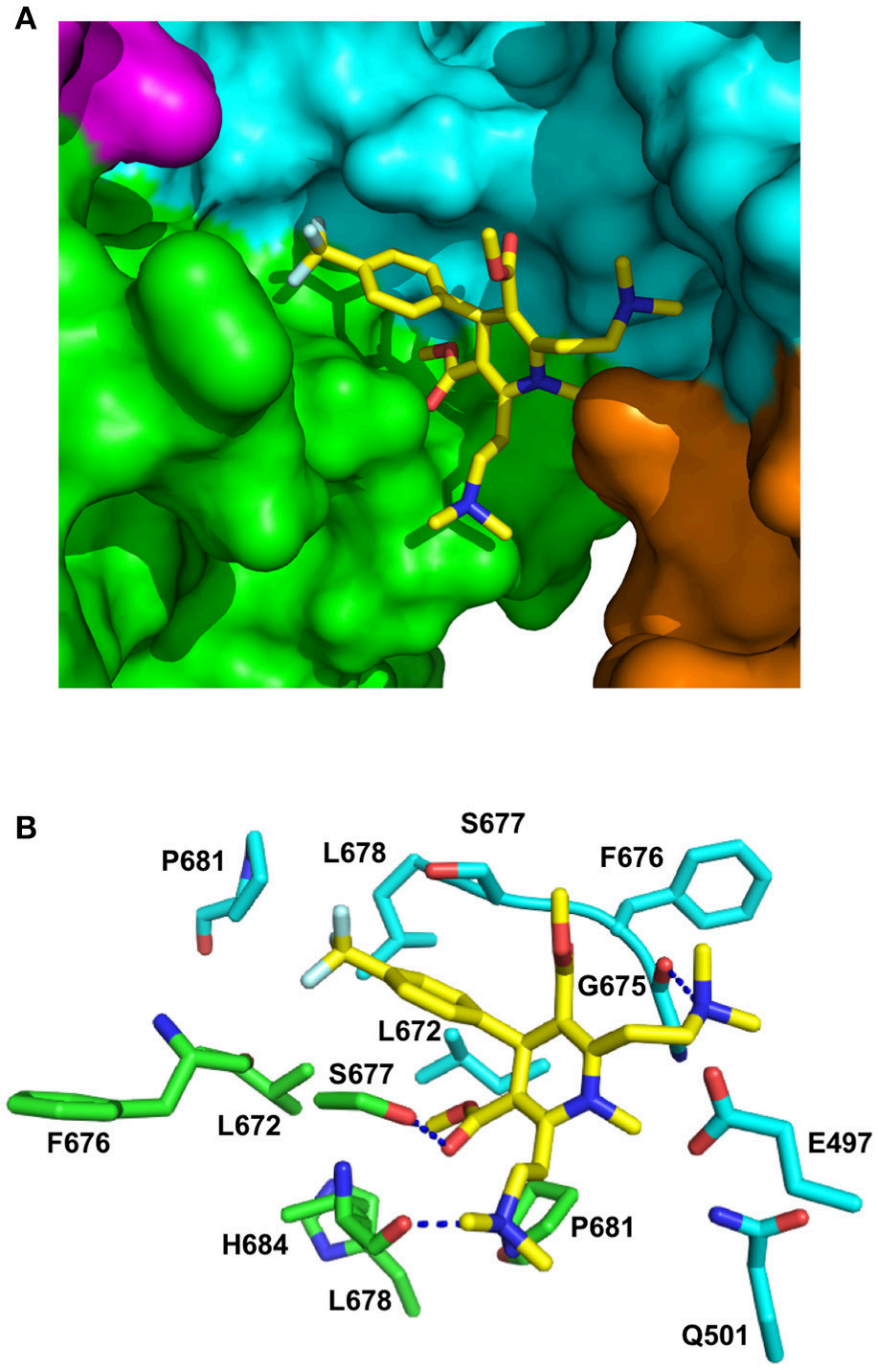
PyMol cartoons of LA1011 bound to Hsp90. **(A)** Surface representation of Hsp90α C-terminal and middle domain regions with LA1011 (yellow sticks) bound at the interface of the two Hsp90 protomers. The protomers of Hsp90 are green-magenta and cyan-gold, respectively. **(B)** PyMol cartoon representation showing the C-terminal and middle domain residues that were identified as potentially interacting residues with LA1011 through molecular dynamics simulations (residue numbers are for Hsp90α). LA1011, yellow sticks; cyan residues, C–terminus and middle domain of Hsp90 protomer 1 and green residues, C–terminus and middle domains of Hsp90 protomer 2. Blue dashes, potential hydrogen bonds.

The stoichiometry for the binding of LA1011 was clearly 0.5:1 (Figure 1), representing one LA1011 molecule per Hsp90 dimer. We used docking studies to test whether this would locate LA1011 into a site upon the human Hsp90α dimer that was consistent with this stoichiometry. We found that LA1011 bound the symmetric dimer of Hsp90 asymmetrically occupying a single site within the dimer (Figure 8). This was consistent with the stoichiometric interaction with Hsp90 determined by ITC (Figure 1).

During the molecular dynamic simulations, as was expected, the hydrophobic head group of the ligand buried itself into a hydrophobic ridge, over a hydrophobic cage motif, with the polar tails being more flexible around the polar side-chains, further from the interface (Figure 8). Overall, LA1011 sits in amongst loops connecting the two terminal helices that form the inherent C-terminal dimer interface of Hsp90, another loop that is formed by the C-terminal residues 601–632, much of which is disordered, and a middle domain loop formed by residues 495–499. The phenyl ring of LA1011 sits exactly at the center of the dimer between Ser 677 from each protomer (Figure 8B). This ensures that only one molecule of LA1011 can bind a single Hsp90 dimer. The trifluoromethyl group projects into a pocket lined by Leu 672, Leu 678, Pro 681, and the main chain of Phe 676. Potential hydrogen bonds are formed between the nitrogen atom of the two ethyldimethylamine groups of LA1011, with either the main-chain carbonyl of Gly 675 or the main-chain carbonyl of Leu 678. The hydroxyl of Ser 677, potentially, also hydrogen bonds the carbonyl group of the methyl methanonate. In contrast, the methyl substituent of the same methyl ethonate group projects into a hydrophobic pocket lined by the side chains of Leu 672 form each protomer, and those of Leu 678, Pro 681, and His 684.

### Mutagenesis supports the C-terminal binding site identified by docking studies

**Figure 9:**
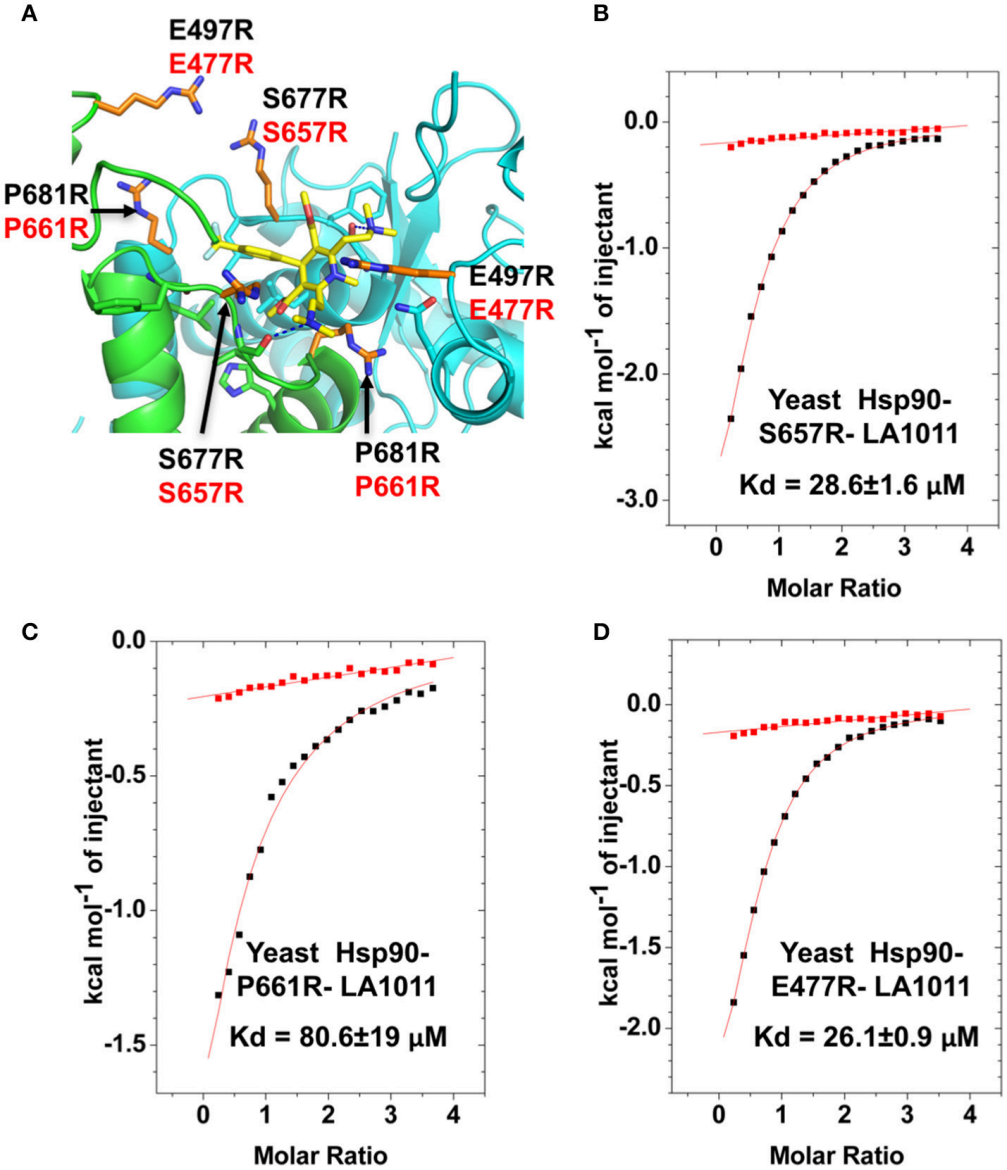
Effect of Hsp90 mutants on LA1011 binding. **(A)** PyMol cartoon showing residues mutated close to the LA1011 binding site and their potential conformation within the structure. Residues mutated to arginine are shown in gold stick representation. Residue numbers in black are for Hsp90α, while those in red are for yeast Hsp90. Yellow sticks, LA1011. **(B–D)** ITC binding curves for LA1011 to the Hsp90 mutants S4657R, P661R, and E477R, respectively. Red lines in ITC figures represent heat of dilution, while the black fittings represent the corrected heat of interaction for the experiment.

In order to validate the C-terminal binding site for LA1011 identified in docking studies we used mutants of Hsp90 that would interfere with the binding of LA1011. Our model did not reveal side chain interactions that if mutated would readily disrupt binding of LA1011. Consequently, we identified residues that could potentially impact on binding by providing slight steric hindrance during binding (Figure 9A). Our analysis suggested that the yeast mutation P661R (P681 in Hsp90α) would be more disruptive than E477R (E487 in Hsp90α) and S657R (S677 in Hsp90α). Using ITC, we found that the *K*d for the binding of LA1011 to these mutants was 80.6 ± 19, 26.1 ± 0.9, and 28.6 ± 1.6 μM, respectively (Figures 9B–D). This is consistent with the predicted effects of these mutations based on our model and confirms that the C-terminal site identified by docking studies is the site for LA1011 binding.

## Discussion

Currently, it is thought that Aβ plaques, formed due to the aggregation of Aβ-precursor protein, trigger the hyperphosphorylation of Tau resulting in neurofibrillary tangle formation and neurotoxicity (Hardy and Selkoe, 2002; Selkoe, 2002; Oddo et al., 2003; Tanzi and Bertram, 2005; Annaert and De Strooper, 2010; Jinwal et al., 2011; Karran et al., 2011; Benilova et al., 2012; Choi et al., 2015). Tau accumulation and aggregation is facilitated by Hsp90 (Blair et al., 2013), and consequently efforts are directed toward developing Hsp90 inhibitors that promote Tau degradation (Dickey et al., 2007; Luo et al., 2007; Blair et al., 2014). The novel DHP-compounds, LA1011 and LA1044, were previously shown to act as co-inducers of Hsp70 and Hsp27 in the SH-SY5Y human neuroblastoma cell line (Kasza et al., 2016). Indeed, LA1011 and LA1044 increase heat shock protein expression in cells that are stressed or diseased. In that study the best co-inducer, LA1011, could protect neurons in the hippocampus, could decrease Tau-accumulation and was able to preserve dendritic spines in the APPxPS1 transgenic mouse model of AD.

Molecular chaperones together with the ubiquitin-proteasome system represent a means by which misfolded proteins in AD could be targeted for clearance (Sulistio and Heese, 2016) and some heat shock proteins show neuroprotective action *in vivo*, (Liu et al., 2009; Manaenko et al., 2010; West et al., 2012; Doyle et al., 2013; Bobkova et al., 2014; Eroglu et al., 2014; Cohen et al., 2015). While unregulated HSP induction could lead to the instability of the HSR (Lamech and Haynes, 2015), heat shock protein co-inducers, like LA1011, that specifically target diseased cells would offer a potentially very useful strategy against neurodegenerative diseases such as AD. Our investigations herein suggest that the molecular mechanism by which LA1011 acts is through the modulation of Hsp90 activity by binding predominantly to its C-terminal domains. It appears that binding to Hsp90 activates its ATPase activity and that these compounds appear to bind Hsp90 to both the open (Sti1 bound) and closed conformation of Hsp90 (Hsp90-AMPPNP). Furthermore, LA1011 binding to the closed Hsp90-AMPPNP-Sba1 complex was only slightly affected.

Our results show that the *K*d for LA1011 binding to Hsp90 is in the region of 3.8–13.5 μM, which correlates with the 5–10 μM levels of LA1011 that had an effect in mouse models (Kasza et al., 2016).

However, effects on the *in vitro* ATPase activity of Hsp90 were much higher. 10 μM levels of LA1011 had marginal effects on the ATPase activity of Hsp90 and levels as high as 240 μM were required to stimulate the ATPase by 2-fold. The reason for this is that nucleotide (ADP and AMPPNP) influences the binding of LA1011 reducing its *K*d from 13 to 66 μM for yeast Hsp90. While this appears to be a minor change, the fractional occupancy of the LA1011 binding site would require 0.5 mM LA1011 to achieve 88% occupancy in the presence of ATP, but only 100 μM in its absence. These levels of LA1011 correlated well with the levels used in the citrate synthase aggregation- and luciferase-assay experiments. However, the difference between *in vivo* levels (5–10 μM) that affect cells and those required (up to 1 mM LA1011) to affect the *in vitro* folding of luciferase remains around 100 times different. Such a difference in the levels of potency of small molecules that target Hsp90, between *in vivo* and *in vitro* experiments, has previously been reported and differences of up to 100-fold were seen (Kamal et al., 2003). It appears that in diseased cells Hsp90's sensitivity toward small molecules that target Hsp90 is much greater, but the exact reasons for this remains enigmatic. Finally, while it is easy to reconcile the effect of inhibition of Hsp90 by compounds such as geldanamycin, which halt chaperone cycle, and thus prevent protein folding. It is, however, not so obvious how small molecules that accelerate the chaperone cycle would have the same effect. Clearly the dwell time of the cycle must influence the productive folding of a client protein and, in fact, this has been previously reported (Zierer et al., 2016).

The mechanism by which the activation of the ATPase activity of Hsp90 occurs is likely to be similar to rhamnoside, which is thought to modulate the conformation of Hsp90 by lowering the transition state that favors N-terminal dimerization and therefore ATPase activity (Sattin et al., 2015). LA1011 is likely to work by a similar mechanism and changes in the chaperones rate might interfere with the mechanism by which client proteins are folded. For example, it has been seen that downregulating the ATPase activity of Hsp90, by displacing Aha1 from Hsp90 complex, can rescue misfolding of the delta F508 mutant CFTR that causes cystic fibrosis. Presumably the mutant CFTR requires longer to fold than wild type CFTR and benefits from a slower chaperone cycle or dwell time (Wang et al., 2006). A model by which proteostasis is maintained by Aha1 regulating the dwell time of Hsp90 with client has been proposed (Koulov et al., 2010). The model suggests that Aha1 activity is able to synchronize chaperone activity with the energetics of client folding by modulating the chaperones ATPase activity. Consequently, LA1011 is likely to be affecting the synchronization of Hsp90 activity with client protein folding. The degree of effect on client folding exerted by an increase in the ATPase activity of Hsp90 might, however, depend on the exact client protein in question. Some clients may be able to tolerate higher rates of Hsp90 activity before their folding is affected, while others may be more sensitive.

Using molecular dynamics simulations and a variety of mutant Hsp90 constructs, we were able to confirm the binding of LA1011 into a pocket formed by a variety of loops from the C-terminal and middle-domains of Hsp90. The mobility of such loops could provide a flexible platform for the binding of a variety of similar compounds that have been shown to bind the C-terminal domain of Hsp90 (Marcu et al., 2000; Donnelly and Blagg, 2008; Lee et al., 2011; Kusuma et al., 2012; Sattin et al., 2015; Vettoretti et al., 2016; Goode et al., 2017), but also the flexibility to provide binding in both the open and closed states of Hsp90. It is also noteworthy that the C-terminal domain of Hsp90 has been implicated as a client protein binding site (Genest et al., 2013) and client protein is known to activate the ATPase activity of Hsp90 (McLaughlin et al., 2002). Consequently, LA1011 may be mimicking the binding of a client protein. The flexibility of this site also raises the possibility that different client protein sequences might be accommodated. However, the structure of Hsp90 in complex with Tau has been determined and it doesn't appear that Tau, at least, interacts with the C-terminal domain of Hsp90 (Karagöz et al., 2014).

Ultimately, whether through competition for client protein binding or through modulation of Hsp90 activity, there is upregulation of the HSR that most likely results from the release of HSF1 from Hsp90 and or because of the higher demand for Hsp90 in diseased or cell stressed states. This, subsequently could be beneficial in AD because of increased levels of Hsp70, Hsp40, and Hsp27 (Heimberger et al., 2013; Blair et al., 2014; Pratt et al., 2015). Furthermore, the hyperphosphorylation of Tau, a known Hsp90 dependent process (Jinwal et al., 2011), may also be disrupted by LA1011, either through direct competition with Tau for binding to Hsp90 or because the Hsp90 cycle is dysregulated leading either to increased E3 ubiquitin ligase dependent degradation or reduced levels of hyperphosphorylation of Tau. However, whichever mechanism is responsible for the disruption of Tau hyperphosphorylation, this in turn translates to a decrease in the accumulation of neurofibrillary tangles and neurotoxicity (Drewes, 2004). Thus, both elevated chaperone levels, which may aid clearance of Aβ plaques, and decreased levels of hyperphosphorylated Tau could be changes in response to LA1011 that are likely to be beneficial to the prognosis of AD.

The C-terminal domain of Hsp90 has been shown to interact with nucleotides and it has been proposed that there is a C-terminal ATP binding site (Marcu et al., 2000; Garnier et al., 2002). However, structural studies have not revealed the presence of an obvious ATP binding pocket capable of hydrolyzing ATP (Ali et al., 2006). It is interesting to note that the binding of LA1011 in the presence of ADP or AMPPNP was identical suggesting that these nucleotides influence and weaken the binding of LA1011 to the same degree. The binding of another Hsp90 C-terminal inhibitor, C095-0286 was recently modeled to the yeast C-terminal binding domain (Jiang et al., 2017). This raises the possibility that the nucleotide and C-terminal LA1011 binding site are one and the same. If so, it might be that this site is an adenine nucleotide binding site acting as a sensor responding to total cellular levels of adenine nucleotide and/or it might act as a regulatory site with which either co-chaperones or client proteins are able to interact with. Further studies will be required to unravel the significance of this observation. Currently, we are trying to determine a co-crystal structure to confirm the molecular details of the LA1011 binding site.

## Author contributions

CP, IH, and LV: conceptualization; CP, MSR, ZT, and BW: methodology; CP and BW: investigations; CP: writing—original draft; CP, IH, LV, and BW: writing—review and editing; CP: supervision.

### Conflict of interest statement

CP and MSR collaborated with Vernalis for the discovery of N-terminal HSP90 inhibitors. The remaining authors declare that the research was conducted in the absence of any commercial or financial relationships that could be construed as a potential conflict of interest.

## Footnotes

^1^The PyMOL Molecular Graphics System, Version 2.0.
